# Continuous Intra-Incisional Bupivacaine for Postoperative Analgesia after Hip Nailing Surgery: A Randomized Clinical Trial

**DOI:** 10.1155/2024/2357709

**Published:** 2024-07-22

**Authors:** Arash Farbood, Sanaz Abbasi, Naeimehossadat Asmarian, Mahsa Banifatemi, Vida Naderi-boldaji, Zeinabsadat Fattahi Saravi

**Affiliations:** Anesthesiology and Critical Care Research Center Shiraz University of Medical Sciences, Shiraz, Iran

## Abstract

**Background:**

The effectiveness of continuous wound infiltration (CWI) as a postoperative pain-control technique has been shown in many surgical procedures. This study investigates the effect of CWI of local anesthetic on postoperative pain control in intertrochanteric fracture patients undergoing hip nailing surgery.

**Methods:**

In this randomized clinical trial, 48 patients who were scheduled for hip nailing surgery were randomly assigned to receive (*n* = 24) or not receive (*n* = 24) bupivacaine infusion through a catheter inside the surgical wound, postoperatively. Pain intensity (NRS), required dose of morphine, and drug-related complications within 24 hours of the intervention were assessed and compared.

**Results:**

Pain intensity was significantly lower in the bupivacaine group both during the recovery room stay and in the ward in the first 24 hours after the procedure (*P* < 0.001). In the recovery room, the control group patients had a higher morphine consumption compared to the bupivacaine group (*P* < 0.001) and requested it earlier than the bupivacaine group (60 (45–60) vs. 360 (195–480) minutes) (*P* < 0.001). In the ward, all control group patients used the PCA morphine pump, while only 54% of the bupivacaine group self-administered morphine through the pump, with a significantly lower total morphine consumption (1 (0–2) vs. 10 (5–14) mg, *P* < 0.001). None of the patients in the bupivacaine group required additional morphine, while 37.5% of the control requested additional morphine (*P*=0.002). Altogether, the control group had a higher total morphine consumption compared to the bupivacaine group in the first 24 hours (10.5 (6–15.5) vs. 1 (0–2) mg, *P* < 0.001).

**Conclusion:**

CWI of bupivacaine helps better pain reduction during the early postoperative hours while it reduces opioid consumption, minimizes nausea and vomiting, and improves patient satisfaction.

## 1. Introduction

Orthopedic patients undergoing surgery often experience the highest incidence of pain compared to other types of operations [1, 2]. Deep incision with considerable tissue dissection and muscle, bone, and vascular exposure following major orthopedic surgeries causes intense nociceptive stimulations of the musculoskeletal tissue and significant postoperative pain [3]. Inadequate management of postoperative pain in orthopedic patients results in delayed recovery time and mobilization and leads to prolonged hospitalization. Additionally, it contributes to increased healthcare costs and reduces patient satisfaction, ultimately impairing their quality of life [4]. To manage this postoperative pain in orthopedic surgeries, various techniques such as intravenous analgesia, epidural analgesia, and peripheral nerve block techniques have been employed.

Systemic analgesic agents like narcotics and nonsteroidal anti-inflammatory drugs are commonly used for pain relief in orthopedic surgeries. However, they are sometimes moderately effective in major orthopedic surgeries and also come with well-documented side effects such as nausea, vomiting, and respiratory depression [5, 6]. Although local anesthetic drugs provide adequate analgesia and reduce the need for systemic medication, frequent painful injections would be harmful since they increase the risk of surgical wound infection and hematoma [7].

Continuous wound infiltration (CWI) using especially designed catheters is a safe and effective alternative to other regional anesthetic techniques like nerve blocks and spinal-epidural anesthesia. The effectiveness of CWI as a postoperative pain-control technique has been revealed in cardiac, thoracic, extensive gynecologic [8], and orthopedic procedures like arthroscopic ACL surgery [9], bone graft extraction [10], spine surgeries [11], and hip arthroplasty [12]. Considering the high incidence of intertrochanteric fracture in the elderly population and the risks associated with using common analgesics (e.g., nonsteroidal anti-inflammatory drugs and narcotics) and because of some age-related comorbidities, more effective postoperative pain-control techniques with fewer adverse effects should be sought.

This study investigates the effect of continuous wound infusion of bupivacaine on postoperative pain control in intertrochanteric fracture patients undergoing hip nailing surgery.

## 2. Methods

This randomized clinical trial was conducted in a tertiary hospital for orthopedic surgery affiliated with Shiraz University of Medical Sciences. It involved intertrochanteric fracture patients scheduled for hip nailing surgery. The study obtained approval from the university Research Ethics Committee (IR.SUMS.MED.REC.1398.99) and was registered in IRCT (IRCT2018092204108N1).

### 2.1. Study Population

Patients aged 50 to 80 years with ASA physical status I and II and intertrochanteric fracture were enrolled, excluding those with a history of uncontrolled seizures, infection at the spinal injection site, coagulation disorders, renal and liver failure, allergic reaction to the study agents, drug dependency, and those unable or unwilling to participate. Eligible patients provided written informed consent and were randomly assigned to either the control (control) or intervention (bupivacaine) groups.

### 2.2. Study Design

Participants underwent preoperative pain score assessments and were instructed on using a PCA pump and a pain assessment tool (numerical rating scale (NRS)). Spinal anesthesia was administered, utilizing hyperbaric bupivacaine 0.5% (12.5 mg) and fentanyl (10 *µ*g) below the L3 intervertebral space with aseptic precautions. At the end of the procedure, in the intervention group, a 15 cm multiorifice tip catheter (InfilteraLong 600, PAJUNK, Geisingen, Germany) [13] was placed subfascially (below the fascia iliaca) by the surgeon, and a 30 mL bolus dose of 0.25% bupivacaine (bupivacaine, Mylan, 100 mg/20 mL vial, Delpharm, France) was injected through the catheter immediately after implantation. The catheter was connected to an elastomeric pump containing 100 mL of 0.25% bupivacaine, providing a constant anesthetic infusion at a rate of 6 mL/hr. No catheter was inserted in the control group. Both groups received intravenous morphine via PCA pumps in the ward, with a 1 mg bolus dose and a 7-minute lockout interval, without a baseline infusion.

### 2.3. Study Assessment

In the recovery room, the time from the end of the operation to the first request for analgesia was recorded. Pain intensity was assessed using the NRS every 15 minutes (at 15, 30, 45, and 60 minutes) while the patient was in a static state. Intravenous morphine was administered as the sole analgesic. For NRS <4, no intervention was given. For NRS between 4 and 7, 1 mg IV morphine was given every 5 minutes until the pain intensity dropped below 4. For NRS >7, 2 mg IV morphine was administered until the pain score dropped below 7, and then managed according to the protocol for NRS between 4 and 7. The total morphine consumption in the recovery room was recorded.

In the ward, pain was managed using a PCA pump loaded with a 1 mg/ml morphine sulfate solution syringe. Additional morphine was given as needed according to the abovementioned protocol. Pain intensity was assessed every 15 minutes in the recovery and every 2 hours within the next 24 hours in the ward. Morphine side effects (respiratory depression, pruritus, urinary retention, nausea, and vomiting) were evaluated every 4 hours. The complications would be managed according to the hospital local protocols. The patient's ability to perform activities such as getting in and out of bed, standing from a chair, and walking was assessed using the Cumulated Ambulation Score.

### 2.4. Statistical Analysis

Data were analyzed using SPSS 21 (IBM, USA) and GraphPad Prism 9 software. Continuous variables were reported as mean ± SD or median (IQR). Independent sample *t*-test and Mann–Whitney *U* test were used for continuous variables. Categorical variables were reported as numbers and percentages, and the chi-square test and Fisher exact test were used for categorical outcome variables. Repeated-measures ANOVA test was utilized for analyzing data over time, with Bonferroni correction applied for pairwise comparisons. Statistical significance was defined as *P* < 0.05.

### 2.5. Sample Size and Randomization

**Figure 1:**
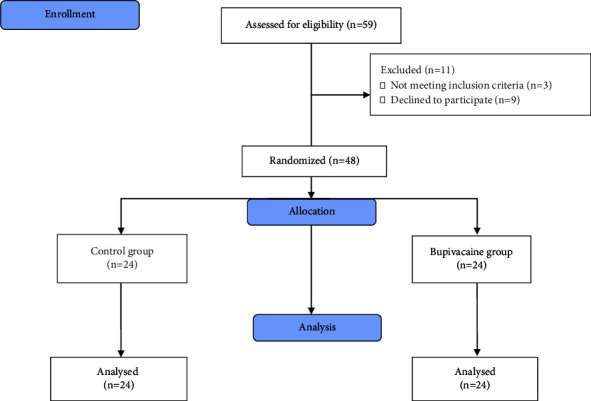
CONSORT flow diagram.

The research involved 24 participants in each group (control = 24 and bupivacaine = 24). It was determined to ensure an 80% power and a type I error of 0.05 in detecting a 20 mm difference in mean NRS scores. It was assumed that the mean NRS score for the bupivacaine group was 30 mm and that for the control group was 10 mm, with a standard deviation of 24 mm. Thus, a minimum of 24 patients per treatment group was required. Participants were assigned to groups using block randomization with 6 blocks of sizes 6 and 8 (block list extract from https://www.sealedenvelope.com) (Figure 1).

## 3. Results

No significant differences were observed between the bupivacaine and control groups with regard to age, sex, weight, BMI, and preoperative pain score (*P* > 0.05), as shown in Table 1.

Table 2 demonstrates the data regarding morphine administration in the bupivacaine and control groups.

In the recovery room, total morphine consumption in the control group was significantly higher than that of the bupivacaine group (4 mg (2.25–5) vs. 0 mg (0-0), *P* < 0.001). Additionally, the control group requested morphine earlier than the bupivacaine group (60 minutes (45–60) vs. 360 minutes (195–480), *P* < 0.001).

In the ward, all control group patients used PCA pump for morphine self-administration, with a median morphine consumption of 10 mg (5–14). In contrast, only 54% of the bupivacaine group received morphine through the pump, with a significantly lower median consumption of 1 mg (0–2) compared to the control group (*P* < 0.001). Moreover, none of the bupivacaine group patients required additional morphine, while 37.5% of the control group requested extra morphine in addition to what they received from the PCA pump (*P*=0.002).

Also, the control group had a higher total morphine consumption compared to the bupivacaine group during the first 24 hours after the procedure (10.5 mg (6–15.5) vs. 1 mg (0–2), *P* < 0.001).

### 3.1. Pain Score in Recovery Room and Ward

**Figure 2:**
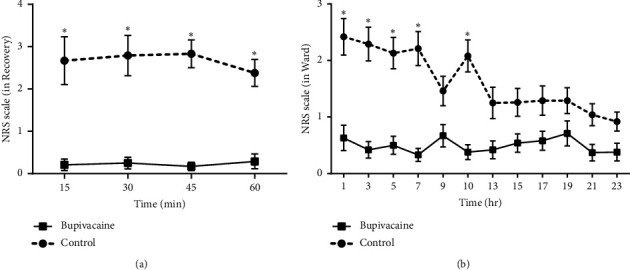
Comparison of NRS scale over time in the studied groups. ^*∗*^Significant *P* value. (a) NRS scale variations during the recovery room stay in both groups of the study. Time effect = 0.77; time^*∗*^group effect = 0.59; group effect <0.001. Bonferroni correction *P* value = 0.012. *P*_times=15,30,45,60_  < 0.001. (b) NRS scale changes in the ward in both study groups. Time effect < 0.001; time^*∗*^group effect < 0.001; group effect <0.001. Bonferroni correction *P* value = 0.004. *P*_times=1,3,5,7,10_  < 0.001.

In the recovery room (Figure 2(a)), the repeated-measures analysis showed that neither the time effect nor the interaction between time and groups was statistically significant (*P*=0.77, *P*=0.59). However, the group effect was significant (*P* < 0.001). It means that throughout the recovery room period, the control group consistently had significantly higher pain levels (NRS) compared to the bupivacaine group.

In the ward (Figure 2(b)), the repeated-measures analysis revealed significant effects for time, the interaction between time and groups, and the group effect (*P* < 0.001). After applying the Bonferroni correction (*P*=0.004) for the 12-time points, it was revealed that at 1, 3, 5, 7, and 10 hours, the pain intensity in the control group was significantly higher than that of the bupivacaine group (*P* < 0.001). Beyond the 10-hour time point, the pain intensity in the control group decreased sharply and although the pain intensity in the control group remained higher than that of the bupivacaine group, this difference was not statistically significant (*P* > 0.004).

### 3.2. Drug-Related Complications

The control group had significantly higher incidence of nausea and vomiting compared to the bupivacaine group (25% vs. 0%, *P*=0.02). However, there were no significant differences between the groups in terms of itching, decreased blood pressure, and urinary retention (*P* > 0.05).

### 3.3. Patient Satisfaction and Movement Ability

The level of patient global satisfaction in the bupivacaine group was significantly higher than that of the control group (87.5% vs. 33.3%, *P* < 0.001). As shown in Table 3, no significant differences were observed between the study groups regarding the patient's independence in three activities assessed using the Cumulated Ambulation Score (*P* > 0.05).

## 4. Discussion

Postoperative pain is a major concern after orthopedic surgeries, impacting patients' physical function and quality of life. In this randomized clinical trial, while the demographic data and preoperative pain scores were matched between the groups, we investigated the effect of continuous wound infusion of 0.25% bupivacaine on reducing postoperative pain in patients undergoing hip nailing surgery for intertrochanteric fractures.

### 4.1. Key Findings

1. The bupivacaine group had a longer time to the first request for analgesia and lower morphine consumption both in the recovery room and in the ward compared to the control group.
2. Throughout the first postoperative 24 hours, patients with continuous bupivacaine infusion had lower mean pain intensity, especially during the recovery room stay and the initial hours in the ward.
3. Bupivacaine infusion increased patients' satisfaction, but there were no significant differences in performing the assessed activities independently between the two groups of the study.
4. Nausea and vomiting were reduced in the bupivacaine group, while itching, hypotension, and urinary retention were similar between groups.

Previous studies have examined the efficacy of continuous local anesthetic infusion in different surgical procedures. In a study of patients undergoing total knee arthroplasty (TKA), Karen Toftdahl et al. detected that intraarticular application of analgesics (ropivacaine (2 mg/mL), 1 mL ketorolac (30 mg/mL), and 1 mL epinephrine (0.5 mg/mL)) significantly decreased pain scores during activity and consumption of opioids on the first postoperative day [14]. Ropivacaine continuous wound injection could efficiently alleviate postoperative pain and decrease blood loss and the length of hospital stay after spine fusion surgery and laparoscopic cholecystectomy [15, 16].

However, our findings contradict previous studies [17, 18] where continuous intra-articular infusion of bupivacaine (0.25%, 4 mL/h) did not provide pain relief or reduce medication use after anterior cruciate ligament reconstruction. This lack of effectiveness may be due to the choice and dosage of the local anesthetic. The flow rate of 4 mL/h may have been insufficient for effective pain relief in a major orthopedic procedure. Improper catheter placement is another possible reason, as it could have prevented bupivacaine from reaching the nociceptors.

In accordance with others [11], except for the incidence of postoperative nausea and vomiting, we observed similar rates of hypotension, itching, and urinary retention in both groups. Furthermore, no cases of respiratory depression occurred. The lower incidence of nausea and vomiting in our study's intervention group might be due to less morphine consumption compared to the control group. It is worth noting that patients with cardiac and respiratory disease are at increased risk for opioid-induced respiratory dysfunction (OIRD) [19], so the likelihood of respiratory complications is lower in patients with ASA I and II, which were the inclusion criteria for our study.

## 5. Conclusion

In conclusion, the efficacy of continuous wound infusion of bupivacaine on postoperative pain control in intertrochanteric fracture patients undergoing hip nailing surgery was tested. Our study showed that continuous wound analgesia injection provides superior pain control, reduces opioid consumption, minimizes nausea and vomiting, and improves patient satisfaction. This therapy could be considered as a part of a multimodal analgesia regimen to alleviate postoperative pain of hip nailing surgery.

## Figures and Tables

**Table 1:** Comparison of demographic and baseline data between the study groups.

	Control *n* = 24	Bupivacaine *n* = 24	*P* value
Age (year)	75.5 (65.75–81.75)	79 (63.75–82)	0.75
Sex, male	16 (66.7)	10 (41.6)	0.08
Weight (kg)	66.67 ± 11.22	67.88 ± 11.52	0.71
BMI	23.69 ± 3.63	23.62 ± 3.49	0.95
Preoperative pain score	6.71 ± 0.74	6.52 ± 0.79	0.39

The values are represented as mean ± SD or numbers (percentages).

**Table 2:** Information about morphine uses of the studied groups in the recovery room and ward.

	Control *n* = 24	Bupivacaine *n* = 24	*P* value
Recovery			
Time to first morphine request (min)	60 (45–60)	360 (195–480)	<0.001^*∗*^
Total morphine consumption (mg)	4 (2.25–5)	0 (0–0)	<0.001^*∗*^
Ward			
PCA pump morphine (mg)	10 (5–14)	1 (0–2)	<0.001^*∗*^
Additional morphine (mg)	0 (0–1)	0 (0–0)	0.001^*∗*^
Total			
Total morphine consumption during the first 24 hours (mg)	10.5 (6–15.5)	1 (0–2)	<0.001^*∗*^

The values are represented as median (Q_1_–Q_3_) or numbers (percentages). ^*∗*^Significant *P* value.

**Table 3:** The degree of movement ability of patients under study after surgery.

	Control *n* = 24	Bupivacaine *n* = 24	*P* value
Change position			
Cannot	4 (16.7)	6 (25)	0.83
With the helper	16 (66.7)	15 (62.5)	
Without the helper	4 (16.7)	3 (12.5)	
Stand			
Cannot	10 (41.7)	7 (29.2)	0.64
With the helper	13 (54.2)	15 (62.5)	
Without the helper	1 (4.2)	2 (8.3)	
Move			
Cannot	11 (45.8)	13 (54.2)	0.65
With the helper	12 (50)	9 (37.5)	
Without the helper	1 (4.2)	2 (8.3)	

The values are represented as numbers (percentages).

## Data Availability

Data are available by contacting the corresponding author through e-mail.

## Additional Points

*Limitation.* Limitations of this study included the following: (1) limited surgical team knowledge of optimal catheter placement; (2) co-location of intra-incisional catheter and Hemovac drain leading to drug wastage; (3) potential catheter damage during dressing changes; and (4) financial constraints to provide intra-incisional catheters for the control group for blinding.
